# Case report of 49,XXXXY syndrome with cleft palate, diabetes, hypothyroidism, and cataracts

**DOI:** 10.1097/MD.0000000000017342

**Published:** 2019-09-27

**Authors:** Limin Wei, Yi Liu, Sufen Sun, Yong Tang, Shuchun Chen, Guangyao Song

**Affiliations:** a Department of Endocrinology and Metabolism, Hebei General Hospital, Shijiazhuang; b HeBei North University, Zhangjiakou, Hebei, China.

**Keywords:** cataracts, diabetes mellitus, Klinefelter syndrome

## Abstract

**Rationale:**

The karyotype 49,XXXXY is a rare form of Klinefelter syndrome usually presenting with ambiguous genitalia, facial dysmorphism, mental retardation, and a combination of cardiac, skeletal, and other malformations.

**Patient concerns:**

We describe a 19-year-old man, whose chromosomal analysis of peripheral blood revealed a karyotype of 49,XXXXY. His mental development and motor ability were significantly delayed. At the age of 19, he had failed to develop secondary sexual characteristics. His random blood glucose level was 19.61 mmol/L, and he showed dry mouth, polydipsia, and polyuria. He had a characteristic facial appearance with prognathism, widened nasal bridge, and strabismus. His bilateral elbow rotation was limited. He had atrophic testes with micropenis. Ophthalmic examination revealed a polar cataract in both eyes.

**Diagnosis:**

He was diagnosed with Klinefelter syndrome associated with cleft palate, hypothyroidism, cataracts, diabetes, and other anomalies.

**Interventions:**

After the initial diagnosis, the patient received intensive insulin therapy to correct hyperglycemia, and he received calcium and vitamin D supplements. The patient also received testosterone and thyroid hormone replacement therapy for primary hypogonadism.

**Outcomes:**

The patient was discharged 12 days after receiving treatment; meanwhile, there were no clinical symptoms of dry mouth, polyuria and polyuria, and his blood glucose level was controlled.

**Lessons:**

The combination of cleft palate, hypothyroidism, cataracts, diabetes, and osteoporosis in 49,XXXXY syndrome has not yet been reported. Early treatment and appropriate care can significantly improve the patient's quality of life and prevent serious consequences.

## 1 Introduction

The genetic background of Klinefelter syndrome is the extra X-chromosome, which may be inherited from either parent. The disease exists with different karyotypes, from 47,XXY to 49,XXXXY. The key findings in Klinefelter syndrome include small testes, hypergonadotropic hypogonadism, and cognitive impairment. The hypogonadism may lead to changes in body composition, and a risk of developing metabolic syndrome and diabetes. The cognitive impairment mainly involves language processing.

49,XXXXY syndrome is a rare sex chromosome aneuploidy syndrome with an incidence of about 1/85,000.^[1,2]^ These patients are frequently diagnosed as having “Klinefelter variant.”^[3]^ Herein, we report a 49,XXXXY Klinefelter syndrome male with cleft palate, hypothyroidism, cataracts, and diabetes. These anomalies have never been previously reported in a single patient.

### 1.1 Consent statement

Written informed consent was obtained from the parents of patient for the publication of this study (Supplemental Digital Content).

## 2 Case report

A 19-year-old man was admitted to our hospital for the treatment of hyperglycemia and cataracts. He was the second child of healthy unrelated parents. There was no family history of chromosomal abnormality. His older sister was healthy with normal development. During infancy, his mental development and motor ability were significantly delayed. Cleft palate repair was performed at 6 years of age. At the age of 19, he had failed to develop secondary sexual characteristics. He was 172 cm and 47 kg, and his sexual development was Tanner stage I.

**Figure 1:**
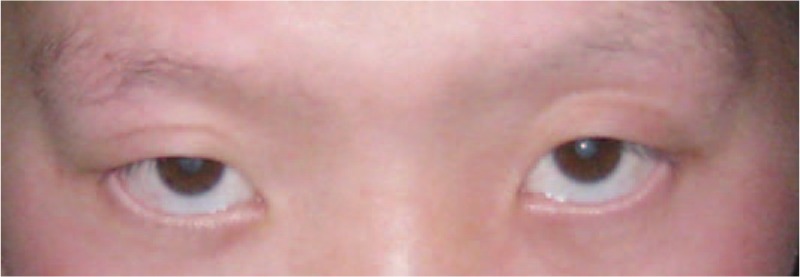
Characteristic facial appearance of the patient with strabismus.

**Figure 2:**
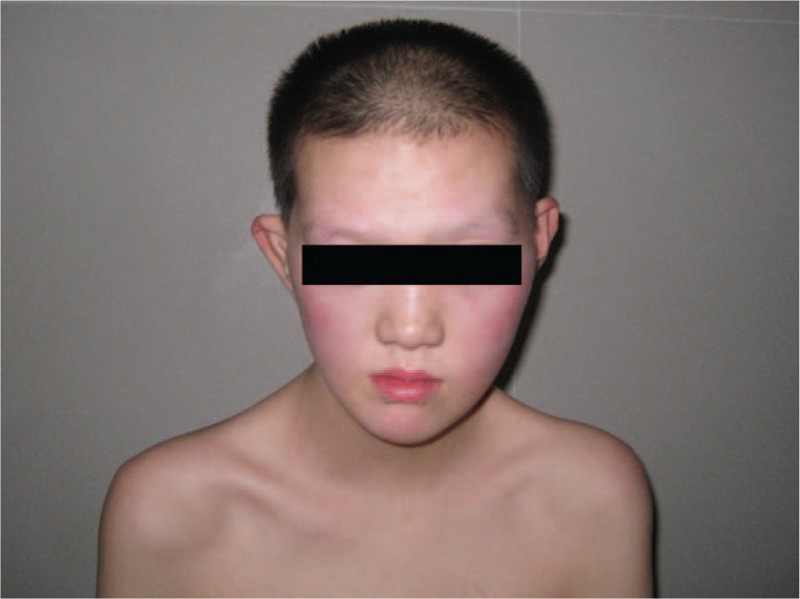
Characteristic facial appearance of the patient with prognathism and widened nasal bridge.

**Figure 3:**
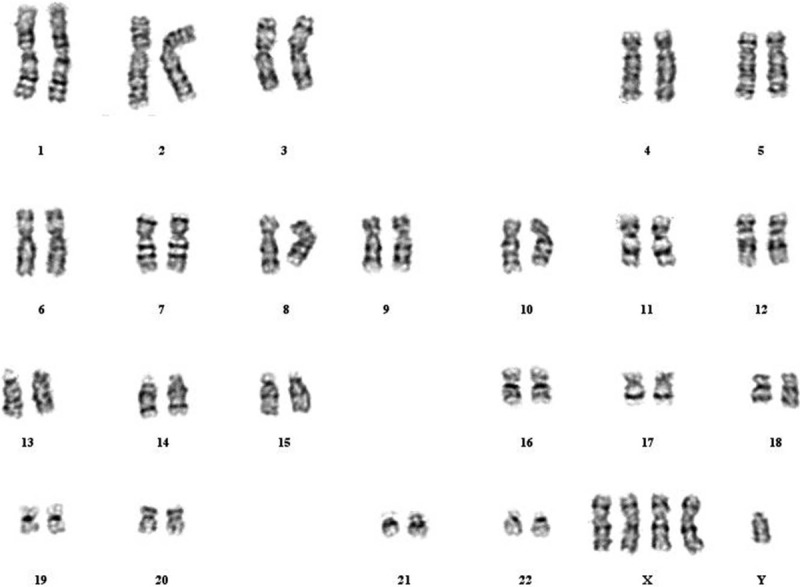
Karyogram of the patient showing the 49,XXXXY karyotype.

The patient's admission was characterized by dry mouth, polydipsia, and polyuria. Random blood glucose level was 19.61 mmol/L. He had a characteristic facial appearance with prognathism, widened nasal bridge, and strabismus (Figs. 1 and 2). Ophthalmic examination revealed a polar cataract in both eyes. His bilateral elbow rotation was limited. He had atrophic testes with micropenis. After signing the informed consent, a peripheral venous blood sample was obtained from the patient for karyotyping. Routine karyotyping was performed on GTG-banded metaphases from cultures of PHA-stimulated peripheral blood lymphocytes according to standard procedures. Cytologic studies on peripheral blood cultures indicated the 49,XXXXY karyotype (Fig. 3).

The patient had increased serum LH at 29.67 IU/L (normal range: 1.70–8.60 IU/L) and FSH at 50.14 IU/L (1.50–12.40 IU/L), but his FT was low at 0.38 ng/ml (2.80–8.00 pg/ml). His TT3, TT4 levels were low at 0.97 nmol/L and 49.08 nmol/L (1.30–3.10 nmol/L and 66.00–181.00 nmol/L), respectively, and TSH level was normal. The HbA1C was 12.2%. The FCP level was 0.80 ng/mL (1.01–4.40 ng/mL). Laboratory investigations, including hemogram, serum cholesterol, urinalysis, blood urea nitrogen, calcium, phosphorus, and alkaline phosphatase levels, were normal. BMD examination showed osteoporosis.

His hyperglycemia necessitated constant intensive insulin therapy, and he received calcium and vitamin D supplements. The patient also received testosterone and thyroid hormone replacement therapy for primary hypogonadism. The patient was discharged 12 days after receiving treatment; meanwhile, there were no clinical symptoms of dry mouth, polyuria and polyuria, and his blood glucose level was controlled.

## 3 Discussion

Klinefelter syndrome (KS) (47,XXY) is the most frequent chromosomal aberration in males,^[3]^ but 49,XXXXY syndrome represents a rare form of Klinefelter syndrome. Boys with this karyotype are assumed to have severe mental retardation in addition to craniofacial, genital, endocrine, and heart abnormalities.^[1]^ The patients with 47,XXY karyotype may be asymptomatic until puberty with similar symptoms, but the patients with 49,XXXXY karyotype can be diagnosed earlier because of severe anomalies such as mental retardation and hypogonadism.^[2,4]^ 49,XXXXY karyotype is thought to arise from maternal nondisjunction during both meiosis I and meiosis II.^[5,6]^

In contrast to other 49,XXXXY syndrome cases, this patient not only had classical clinical features of the syndrome but also cleft palate, hypothyroidism, cataracts, diabetes, and osteoporosis. Cleft palate, one of the anomalies that is occasionally seen in the 49,XXXXY Klinefelter variant, is not pathognomonic.^[7]^ A patient with cleft palate must be considered to have a variant and not the classic form of Klinefelter syndrome. Another report showed 49, XXXXY syndrome patients with external genital hypoplasia, subclinical hypothyroidism, and severe scoliosis.^[8]^ Meanwhile, Kim et al^[9]^ reported an 18-year-old patient with 49,XXXXY syndrome accompanying diabetes mellitus. The incidence of diabetes among patients with Klinefelter syndrome is higher than normal.^[10]^ However, the reported cases of diabetes in patients with 49,XXXXY syndrome are rare.^[11]^ The reason for the small number of diabetes mellitus cases among patients with 49,XXXXY is uncertain. Cataract, which is associated with chromosomal anomalies, is usually congenital that is diagnosed at infancy and early childhood.^[12]^ Our patient did not suffer from congenital cataract, so we considered that the cataract in this patient was perhaps related to diabetes. However, the association of these features in 49, XXXXY syndrome patients as described herein has not been reported.

This syndrome had a general shift toward lower values in distribution of serum FT4 with no compensatory increase in serum TSH.^[9,13]^ Decreased BMD and osteoporosis affecting morbidity and mortality have been linked to Klinefelter syndrome.^[3]^ The low bone mass is readily explained by hypogonadism, resulting in low physical exercise capacity and muscle strength, and treatment with testosterone can improve BMD, and the changes in thyroid functioning may also exacerbate low BMD.^[13,14]^ Early treatment of Klinefelter syndrome with testosterone can significantly improve the patient's quality of life and prevent serious consequences. Appropriate care for patients with 49,XXXXY syndrome can improve their quality of life.^[15,16]^

The 49,XXXXY syndrome patient described in this article is adult. Because the patient was hospitalized for a short period of time (the family asked to be discharged after 12 days of treatment), so there was a lack of further diagnostic tests and targeted treatments in this case. However, it was gratifying that the treatment plan described in this case can effectively control his abnormally elevated blood glucose level and significantly improve the clinical symptoms of diabetes (such as dry mouth, polydipsia, and polyuria).

## Author contributions

**Conceptualization:** Guangyao Song.

**Data curation:** Yi Liu, Sufen Sun, Yong Tang, Shuchun Chen.

**Project administration:** Limin Wei.

**Supervision:** Limin Wei, Guangyao Song.

**Validation:** Limin Wei, Sufen Sun, Shuchun Chen.

**Writing – original draft:** Limin Wei, Sufen Sun, Yong Tang.

**Writing – review & editing:** Limin Wei, Yi Liu, Yong Tang, Shuchun Chen.
